# Association of gut microbiota and immune gene expression with response to targeted therapy in BRAF mutated melanoma

**DOI:** 10.1038/s41598-025-11054-2

**Published:** 2025-07-14

**Authors:** Mora Guardamagna, Miguel-Angel Berciano-Guerrero, Rocío Lavado-Valenzuela, Édouard Auclin, Juan Luis Onieva-Zafra, Isaac Plaza-Andrades, Javier Oliver, Alicia Garrido-Aranda, Elisabeth Perez-Ruiz, Martina Álvarez, María Carmen Ocaña, María Isabel Queipo-Ortuño, Isabel Barragán, Antonio Rueda-Dominguez

**Affiliations:** 1 Virgen de la Victoria University Hospital, Instituto de Investigación Biomédica de Málaga y Plataforma en Nanomedicina-IBIMA Plataforma BIONAND, 29010 Málaga, Spain; 2 Department of Medicine and Dermatology, Medical School University of Málaga, Campus Teatinos, Blvr. Louis Pasteur, 32, 29010 Málaga, Spain; 3 Department of Cancer Medicine, Gustave Roussy, Villejuif, France; 4 Group of “New Horizons for Cancer Patients” (B-23). Regional University Hospital, Instituto de Investigación Biomédica de Málaga y Plataforma en Nanomedicina-IBIMA Plataforma BIONAND, 29010 Málaga, Spain; 5 Cancer Molecular Biology Laboratory, CIMES, Málaga, Spain; 6 Institut Bergonié, Bordeaux, France; 7 Pathological Anatomy Department, Medical School University of Málaga, Campus Teatinos, Blvr. Louis Pasteur, 32, 29010 Málaga, Spain; 8 Department of Surgical Specialties, Biochemical and Immunology, Faculty of Medicine, University of Málaga, 29071 Málaga, Spain; 9 Group of Pharmacoepigenetics, Department of Physiology and Pharmacology, Karolinska Institute, 171 65 Stockholm, Sweden; 10 Group of Translational Research in Cancer Immunotherapy and Epigenetics (B-05), Medical Oncology Unit of Virgen de la Victoria Hospital, Instituto de Investigación Biomédica de Málaga y Plataforma en Nanomedicina–IBIMA Plataforma Bionand, 29010 Málaga, Spain; 11 Onoc-hematology Unit CIMO - Clinical and Translational Research in Cancer, Instituto de Investigación Biomédica de Málaga y Plataforma en Nanomedicina-IBIMA Plataforma BIONAND, Málaga, Spain

**Keywords:** Gut microbiota, Metastatic melanoma, Immune expression genes, Translational research, BRAF mutated melanoma, Immune system, Skin cancer, Melanoma, Bacteria, Cancer, Immunology, Microbiology

## Abstract

Gut microbiota has been associated with carcinogenesis and immune regulation. While there is evidence supporting its influence on immunotherapy response in melanoma, its impact on BRAF/MEK-targeted therapy remains unexplored. This study assessed gut microbiota composition and immune-associated genes in melanoma, to generate hypothesis on prognostic and predictive biomarkers for BRAF/MEK inhibitor therapy. This prospective, observational study analyzed fecal and blood samples from 26 patients diagnosed with *BRAF*-mutated metastatic melanoma. Samples were collected at baseline (T0) and at first radiological evaluation (T1). Patients were stratified by treatment response and toxicity. Partial responders had baseline enrichment of *Lachnospiraceae*, *Coriobacteriaceae*, and *Adlercreutzia.* Complete responders showed abundance of *Prevotellaceae*,* Cerasicoccaceae* and *Lawsonia*. *Oscillospira* was associated with moderate-to-severe toxicity. Gene expression analysis revealed upregulation of *TAP1* y *PSMB8* during treatment in responders, with persistent downregulation of *LAG3*, *CD36*,* SLAMF7*,* NOD1*,* SLAMF6*,* CX3CR1* and *ICAM2*. Overall, baseline microbiota composition differed by response depth, with abundance of *Lachnospiraceae*, *Coriobacteriaceae*, and *Adlercreutzia* in partial responders and *Prevotellaceae*, *Cerasicoccaceae*, and *Lawsonia* in complete responders. A gene signature of immune-suppresive genes was persistently downregulated in responders. Our findings suggest that specific gut microbial taxa and immune-related gene expression patterns may be associated with differential responses to BRAF/MEK-targeted therapy in metastatic melanoma.

**Supplementary Information:**

The online version contains supplementary material available at 10.1038/s41598-025-11054-2.

## Introduction

Gut microbiota (GM) has emerged as a compelling area of research in oncology, driven by accumulating evidence linking it to carcinogenesis, the tumor microenvironment (TME), and the immune system. It has been shown to regulate immune responses by stimulating antigen presenting cells, CD4 + and CD8 + T cells, and contributing to cytokine secretion, neutrophil migration and function, and T cell differentiation^1^. Consequently, GM has been proposed as a biomarker of treatment response across various modalities, including chemotherapy, radiotherapy, and immune checkpoint inhibitors (ICI)^2^. However, its interaction with targeted therapies, such as tyrosine kinase inhibitors (TKIs), remains largely unexplored.

Melanoma, a highly immunogenic malignancy, harbors *BRAF* mutations in 40–60% of cases, which are associated with poor prognosis^3^. While BRAF/MEK inhibitors (iBRAF/MEK) and ICIs have significantly improved patient outcomes, resistance frequently develops, underscoring the urgent need for novel biomarkers^4^. A promising framework to understand resistance mechanisms integrates three key concepts: melanoma-related immunoediting^5^, the influence of microbiota on treatment responses^6–10^, and the interplay between therapies and the immune system^11^.

Immunoediting describes the dynamic interactions through which the immune system attempts to eradicate cancer cells, exerting selective pressure that can result in the emergence of resistant melanoma cell variants. This process often culminates in immune exhaustion, facilitating tumor evasion from immune surveillance and challenging both immunotherapy and targeted therapeutic approaches^5^. In this context, the microbiota has demonstrated immunomodulatory effects in ICI-treated patients, modulating regulatory T-cells, CD8 + and CD4 + T-cells, myeloid-derived suppressor cells and macrophages^1,12^. While the interaction between GM and targeted therapies in melanoma is underexplored, early evidence suggests that BRAF inhibitors may promote immune activation by reducing immunosuppressive molecules and cytokines within the TME and enhancing CD8 + T-cell cytotoxicity^13,14^. MEK inhibitors have also been shown to modulate immunity by increasing T-cell infiltration and attenuating proinflammatory cytokines, such as tumor necrosis factor^11,15^. This intricate interplay between the immune system, the microbiota, and targeted therapies represents a promising avenue for investigating resistance mechanisms and therapeutic optimization.

Based on these data, we hypothesize that GM composition may influence responses to treatment or toxicity in patients receiving iBRAF/MEK therapy. Specific microorganisms may serve as prognostic or predictive biomarkers of response, as well as of treatment-related adverse events. Additionally, genes associated with immune activation could further refine prognostic and predictive models. By identifying biomarkers for early prediction of response and microorganisms linked to treatment efficacy and toxicity, our findings aim to contribute to the development of microbiota-modulation strategies that could enhance therapeutic outcomes in specific contexts.

## Patients, materials and methods

### Study design

A prospective, observational study was coordinated and carried out in the Regional and Virgen de la Victoria University Hospitals and Institute for Biomedical Investigation (IBIMA) in Málaga, Spain. The study was approved by the Research Ethics Committee of Málaga on May 30th, 2019, and 26 patients were recruited from May 2019 until November 2022.

As the first study to specifically investigate the influence of gut microbiota and immune gene expression on treatment outcomes in patients with *BRAF*-mutated metastatic melanoma receiving iBRAF/MEK, this work was designed as an exploratory, hypothesis-generating study. Given the lack of prior data to inform effect sizes in this setting, a formal sample size calculation was not applicable.

### Patient selection and sample collection

Patients over 18 years old, diagnosed with *BRAF* V600-mutated unresectable or metastatic melanoma - stage IIIC, IIID or IV according to the American Joint Committee on Cancer (AJCC) 8th edition TNM -, and eligible for first-line treatment with iBRAF/MEK were included. Treatment regimens consisted of either dabrafenib (150 mg every 12 h) plus trametinib (2 mg once daily), or encorafenib (450 mg once daily) plus binimetinib (45 mg every 12 h), administered per standard dosing guidelines, until progressive disease or unacceptable toxicity. Previous diagnosis of cancer in the past two years, uncontrolled autoimmune or infectious disease, or inability to provide samples were exclusion criteria. All patients provided written informed consent prior to inclusion. The study was conducted in accordance with the Declaration of Helsinki.

Stool and blood samples were collected before treatment (baseline - T0) and at the first radiological evaluation − 3 months - or progression, whatever happened first (T1). The samples were delivered to the processing laboratory in less than 24 h to guarantee quality. Samples were stored in a -80 C monitored freezer until their processing.

### Disease characteristics and response assessment

Patient demographics, clinicopathologic characteristics, and hematological parameters were analyzed. Patients were monitored every four weeks, or more frequently when clinically indicated, with treatment response assessed via computed tomography scans conducted between weeks 10 and 14. Treatment response was evaluated using the *Response evaluation criteria in solid tumors* (RECIST v1.1).

For immune expression analysis, the cohort was stratified into two groups: Responders (R), comprising patients who achieved a complete response (CR) or partial response (PR), and Non-Responders (NR), including those with stable disease (SD) or progressive disease (PD). In contrast, for GM analysis, due to the smaller number of NR, a dichotomous comparison between responders (CR + PR) and non-responders (SD + PD) was not statistically feasible. To address this limitation while exploring microbial patterns associated with treatment efficacy, we conducted two separate subgroup analyses: (1) complete responders (CR) versus non-complete responders (No-CR), and (2) partial responders (PR) versus non-partial responders (No-PR). These stratifications allowed us to evaluate distinct microbial signatures potentially associated with CR or PR, respectively, while minimizing statistical bias due to group imbalance. This strategy was not only methodologically sound given the group distribution, but also clinically relevant, considering that CR is the subgroup with the most favorable survival outcomes, while PR represents the largest proportion of responders to targeted therapy^16^. Toxicity related to iBRAF/MEK was graded using the *Common Terminology Criteria for Adverse Events* (CTCAE v5.0).

### Statistics of clinical variables

Statistical analyses were conducted using SPSS^®^ 29.0.1.0 and R studio version 4.3.2. A descriptive analysis was carried out for clinical variables. Median and range were calculated for numerical variables, and absolute and relative frequencies for categorical variables. Overall survival (OS) was defined as the time from treatment initiation to death from any cause or latest follow-up, and progression-free survival (PFS) as the time from treatment initiation to documented disease progression or latest follow-up. Survival curves were estimated using the Kaplan–Meier method.

Univariate and multivariate Cox-proportional hazards model was performed to evaluate variables impacting PFS and OS, with results presented as Hazards Ratio (HR) and their 95% Confidence interval (95% CI). Variables included in the multivariate analysis were selected based on their potential impact on survival, including use of antibiotics during targeted therapy, grade ≥ 2 treatment-related adverse events (TRAE), and microbiota enriched in responder patients. Model diagnostics were carefully reviewed to confirm adherence to assumptions, including the proportional hazards assumption, despite the limited sample size. A p value of less than 0.05 was the threshold for statistical significance.

### Fecal microbiota analysis

The GM studies were performed using bacterial DNA isolated from patients’ fecal samples. DNA isolation was performed with a column-based purification kit, and quality control was ensured via capillary electrophoresis (Bioanalyzer^®^). Amplification of hypervariable regions of the bacterial 16 S ribosomal RNA (rRNA) was achieved using primers targeting the V2-4-8 and V3-6, 7–9 regions, in accordance with the Ion16S™ Metagenomics Kit manufacturer’s protocol. Library preparation was conducted using the Ion Plus Fragment Library Kit, with barcode identifiers added via the Ion Xpress Barcode Adapters 1–96 Kit (Thermo Fisher Scientific). Libraries were purified using AMPure^®^ XP beads (Beckman Coulter) before emulsion preparation and sequencing. Sequencing was performed on the Ion Torrent S5™ platform, utilizing the Ion Chef system, Ion 520™/530™ Kit-Chef, and Ion 520™ Chip Kit.

Microbial ecology analysis was conducted with the QIIME2 bioinformatics platform. Operational Taxonomic Units (OTUs) were assigned to sequences based on similarities using the UCLUST algorithm, and taxonomy identification was performed by comparing sequences to the *GreenGenes* database.

Alpha diversity was calculated using Shannon and Simpson diversity indexes, and beta diversity with non-metric multidimensional scaling (NMDS) plot based on Bray-Curtis dissimilarities. Differential microbial taxa were identified using LEfSe (Linear discriminant analysis effect size), which combines the non-parametric Kruskal-Wallis test with linear discriminant analysis to detect significant microbial differences in response to treatment and according to the presence of grade ≥ 2 toxicity. This toxicity threshold was chosen to include clinically meaningful adverse events, representing moderate-to-severe toxicity that may require medical intervention. Corrections for multiple independent comparisons were applied to the LEfSe analysis to ensure statistical rigor.

### Immune reactivation-gene expression analysis

Peripheral blood samples were processed to extract RNA using the RNAEasy Plus Universal Mini Kit™ for frozen samples and the QIAamp RNA Blood Mini Kit™ for fresh samples. RNA quantity and purity were assessed using Nanodrop™, and samples not meeting quality standards were excluded from further analysis.

Differential gene expression analysis was conducted on blood samples collected at T0 and T1. Data were generated using the NanoString nCounter^®^ system with the nCounter PanCancer Immune Profiling Panel™, which evaluates the expression of 770 genes associated with immune cells, tumor antigens, and both adaptive and innate immune responses. Data processing, analysis, and visualization were performed using Nanotube. Normalization of gene expression data was achieved with the RUVg (Remove unwanted variation using control genes) method, and quality control checks were performed using nSolver™. Final statistical analyses were carried out with Limma™ (Linear Models for Microarray and RNA-Seq), stratifying the cohort according to response. Fold changes in gene expression were calculated on a logarithmic scale (log₂), with changes exceeding a threshold of one considered significant. Since the analysis was conducted using a predefined gene panel rather than the entire transcriptome, even small changes in fold change, such as one, can be biologically relevant. The selection of these genes is based on their potential importance in the context of the study, increasing the likelihood that modest changes have meaningful functional significance. In addition, the study was conducted using blood samples with bulk RNA analysis, which combines expression signals from multiple cell types. In this context, cellular heterogeneity can dilute specific expression changes. A seemingly small fold change may therefore reflect more pronounced changes in relevant subpopulations of cells that, when averaged across the bulk sample, result in a modest overall fold change in the final analysis. Longitudinal analyses comparing gene expression between T0 and T1 were conducted using the Wilcoxon signed-rank test. A p-value of < 0.05 was considered statistically significant for all analyses.

## Results

### Study population and clinical characteristics

A total of 26 patients with unresectable or metastatic melanoma, eligible for first-line treatment with iBRAF/MEK, provided informed consent to participate in the study. Among these, 20 (76.9%) provided paired fecal samples at T0 and T1 for GM analysis under optimal conditions, while 24 (92.3%) provided both blood samples for gene expression analysis. All GM cohort patients had matching blood samples for gene expression analysis. Two patients who did not provide samples for either GM or gene expression analysis were included in descriptive and survival analyses. Table 1 summarizes the clinicopathological characteristics of the cohort.

Median age was 55 years (range 23–80), with 69.2% patients (*n* = 18) diagnosed before the age of 60. Primary tumor resection was performed in 88.5% (*n* = 23), and 9 had received prior adjuvant treatment - mainly ICI. A total of 21 patients received dabrafenib and trametinib, and 5 received encorafenib and binimetinib. At targeted therapy initiation, 16 (61.5%) patients had stage M1c-d disease.

The overall rate of TRAEs was 92.3% (*n* = 24), with 46.2% (*n* = 12) experiencing grade ≥ 2 events and 23.1% (*n* = 6) experiencing grade 3–4 events. The most common grade ≥ 2 TRAEs were pyrexia (33.3%, *n* = 4), asthenia (16.7%, *n* = 2), and diarrhea (16.7%, *n* = 2). Dose reductions were required in 38.5% (*n* = 10) of patients due to toxicity, and 15.4% (*n* = 4) discontinued treatment as a result of severe adverse events.

**Table 1:** Baseline characteristics, treatment response and survival of intention-to-treat cohort.

Study population = 26 n (%)	
Clinical variables	
Age at stage IV diagnosis, median (range)	55 (23–80)
Sex	
Male	13 (50)
Female	13 (50)
ECOG-PS	
0–1	25 (96.2)
2	1 (3.8)
Antibiotics	
30 days previous treatment	3 (11.5)
During treatment	13 (57.7)
Corticosteroids	
30 days previous treatment	9 (34.6)
During treatment	18 (69.2)
Primary melanoma resected	23 (88.5)
Adjuvant treatment	9 (34.6)
Immunotherapy	8 (30.8)
Interferon	1 (3.8)
Stage M1 (TNM AJCC 8th ed)	
M1a	6 (23.1)
M1b	4 (15.4)
M1c	7 (26.9)
M1d	9 (34.6)
Analytical variables	
LDH ≥ ULN	10 (38.5)
ANC ≥ 7500/mL	5 (19.2)
NLR ≥ 5	7 (26.9)
Severe derived NLR (> 3)	8 (30.8)

Study population = 26 n (%)	
Treatment and toxicity	
BRAF/MEK inhibitor	
Dabrafenib-Trametinib	21 (80.8)
Encorafenib/Binimetinib	5 (19.2)
Toxicity, any grade	24 (92.3)
Pyrexia	9 (34.6)
Asthenia	9 (34.6)
Diarrhea	8 (30.8)
Nausea/Vomiting	8 (30.8)
Hyporexia	3 (11.5)
Rash	2 (7.7)
Myalgias	1 (3.8)
Hyponatremia	1 (3.8)
Hypertransaminasemia	1 (3.8)
Uveitis	1 (3.8)
Neutropenia	1 (3.8)
Toxicity grade ≥ 2	12 (46.2)
Toxicity grade 3–4	6 (23,1)
Pyrexia	2 (7.7)
Asthenia	1 (3.8)
Hepatitis	1 (3.8)
Neutropenia	1 (3.8)
Hyponatremia	1 (3.8)
Dose reduction	10 (38.5)
Discontinuation due to toxicity	4 (15.4)
Response to treatment	
RECIST Response	
Complete response	6 (23.1)
Partial response	14 (53.8)
Stable disease	4 (15.4)
Progressive disease	2 (7.7)
Objective response rate (95% CI)	76.9 (56.4–91.0)
Events	
Progression	14 (53.8)
Exitus	10 (38.5)
Survival	
PFS, median in months, (95% CI)	15.7 (6.0–25.4)
OS, median in months, (95% CI)	28.9 (19.1- 38.7)

ECOG-PS, Eastern Cooperative Group Performance Status; LDH, Lactate dehydrogenase; ULN, Upper limit of normal; ANC, Absolute neutrophil count; NLR, Neutrophil-to-lymphocyte ratio; RECIST, Response Evaluation Criteria In Solid Tumors; PFS, Progression-free survival; OS, Overall survival; 95%CI, 95% Confidence interval

### Treatment response and survival

With a median follow up of 24.3 months, median OS from start of iBRAF/MEK was 28.9 months (IC 95% 19.1–38.7) and median PFS was 15.7 months (95% CI 6.0-25.4). An objective response was seen in 76.9% of patients (*n* = 20), with a total of 23.1% (*n* = 6) achieving CR. A total of 14 (53.8%) patients had progressed to targeted therapy at data cut-off, with 9 (45%) developing central nervous system metastases. Following progression, only 7 patients (35%) were eligible to continue second-line treatment. Univariate analysis did not identify any significant clinical or biological variables associated with survival outcomes, based on the examined factors (Supplementary Table S1).

### Gut microbiota profile in patients treated with targeted therapy

#### Gut Microbiota abundance and diversity according to response

**Fig. 1:**
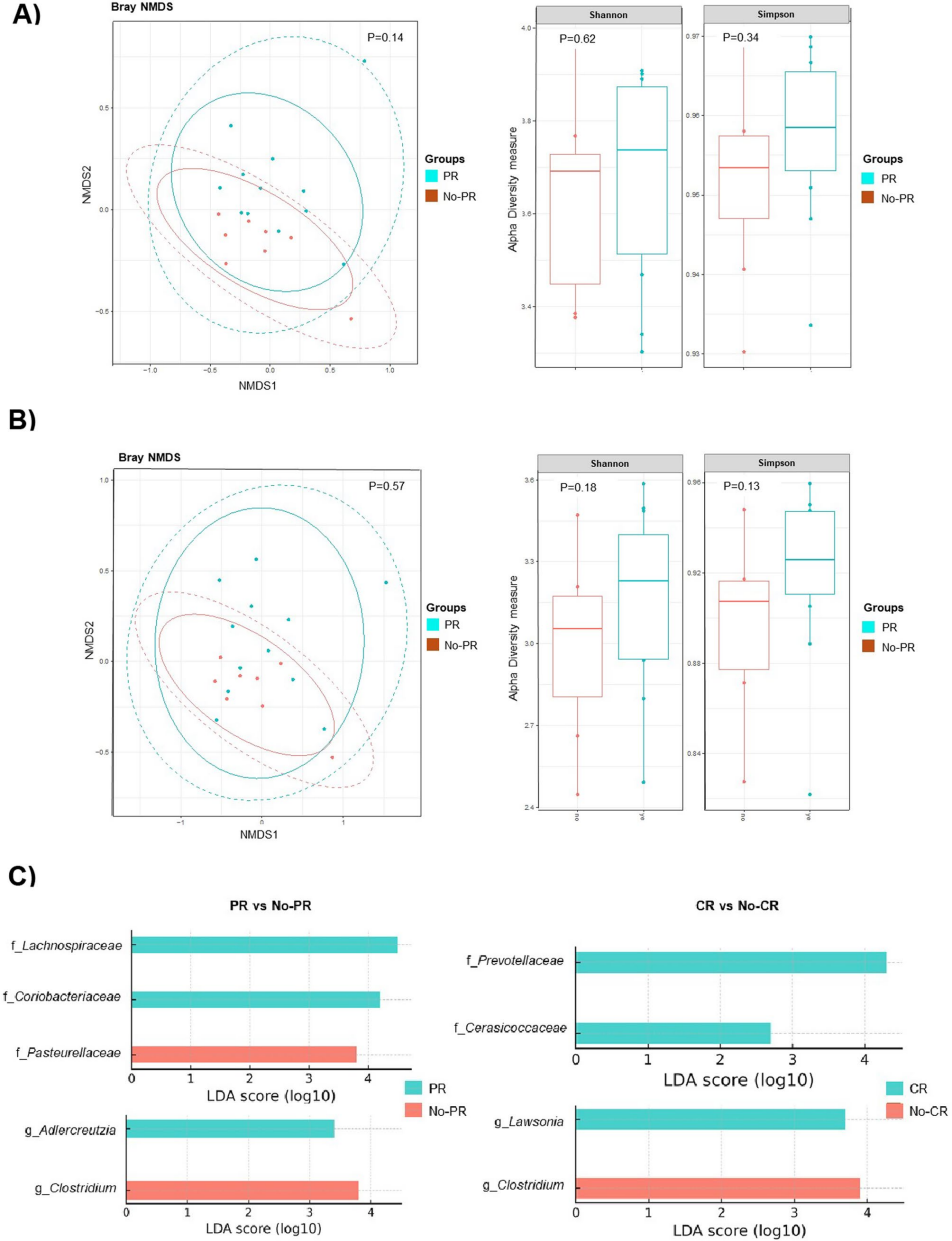
Alpha and beta diversity with a trend towards increased alpha and beta diversity in the partial responder (PR) cohort versus No-PR, though not statistically significant, at (**A**) family level and (**B**) genus level. (**C**) Baseline LEfSe analysis, showing family and genus taxonomy levels, according to response. The presence of each family (f) and genus (g) on the LEfSe denotes statistical significance (*p* < 0.05). PR = Partial response, No-PR = No partial response, CR = Complete response, No-CR = No complete-response.

The GM cohort (*n* = 20) consisted of 5 CR (25%) and 12 PR (60%). No statistically significant differences were observed in alpha or beta diversity when comparing subgroups, though PR demonstrated a trend towards increased alpha diversity (Fig. 1A and B).

At baseline, CR patients exhibited enrichment of families *Prevotellaceae* and *Cerasicoccaceae*, and genus *Lawsonia *(Fig. 1C and Supplementary Figure S1). PR patients showed a significant enrichment of taxa from the Firmicutes and Actinobacteria phyla, with an increased abundance of the *Lachnospiraceae* and *Coriobacteriaceae* families, as well as the genus *Adlercreutzia*. Conversely, No-PR patients were enriched with *Pasteurellaceae* family, while both No-CR and No-PR groups exhibited increased abundance of genus *Clostridium* (Fig. 1C). No statistically significant differences were seen in the longitudinal analysis from T0 to T1 among subgroups.

A multivariate Cox proportional hazards model evaluated the impact of GM composition, TRAE, and antibiotic use on OS and PFS (Supplementary Table S2). However, none of these variables emerged as significant predictors, likely due to the limited sample size.

#### Gut microbiota abundance and diversity according to toxicity

**Fig. 2:**
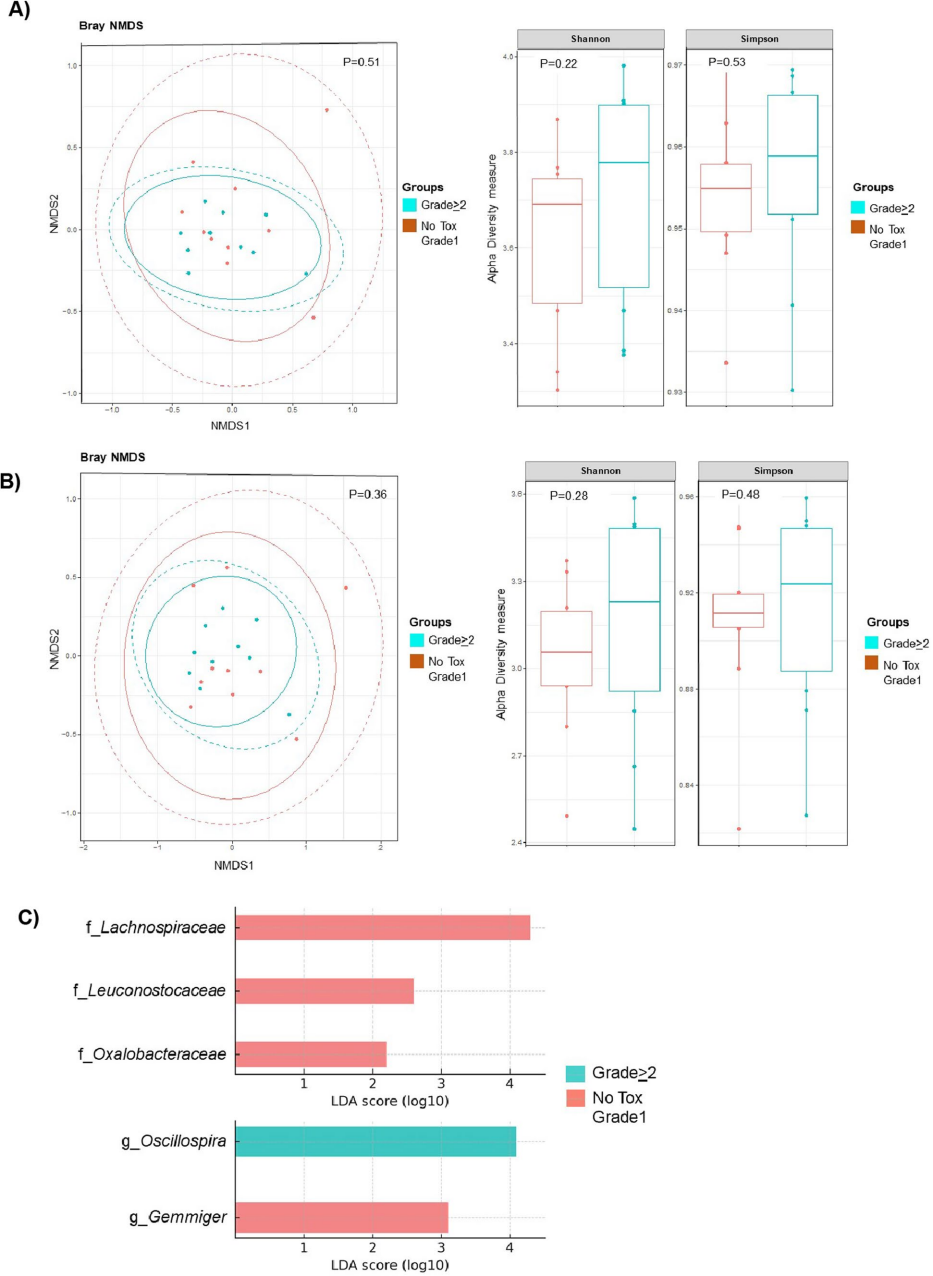
Cohort stratification according to the occurrence of treatment-related adverse events equal or over grade 2. Alpha and beta diversity in (**A**) family and (**B**) genus, with no significant differences among patients that had toxicity equal or over grade 2, compared to no toxicity or grade 1 patients. (**C**) LEfSe analysis, showing family and genus taxonomy levels, according to toxicity. The presence of each family (f) and genus (g) on the LEfSe denotes statistical significance (*p* < 0.05).

GM composition was analyzed by comparing patients who experienced grade ≥ 2 toxicities during targeted therapy (*n* = 10) to those with no or grade 1 toxicities (*n* = 10). No statistically significant differences in alpha or beta diversity were observed between the groups (Fig. 2A and B). However, distinct bacterial families were enriched in each cohort. Bacteria from families *Lachnospiraceae*, *Leuconostocaceae*, and *Oxalobacteraceae*, as well as the genus *Gemmiger*, were significantly overrepresented in patients with no toxicity or grade 1 TRAE. In contrast, only one genus, *Oscillospira*, was enriched in the patients who experienced grade ≥ 2 toxicities (Fig. 2C).

### Immune gene expression profile of response to targeted therapy

Peripheral blood lymphocyte RNA from 24 patients was analyzed to assess the expression of immune-related genes. Among these, 18 patients (75%) were classified as R, including 5 (27.7%) CR, while 6 patients (25%) were classified as NR to the treatment. Normalization of gene expression data successfully eliminated variability and confirmed the absence of batch effects (Supplementary Figures S2 and S3).

#### Stratified by response

**Fig. 3:**
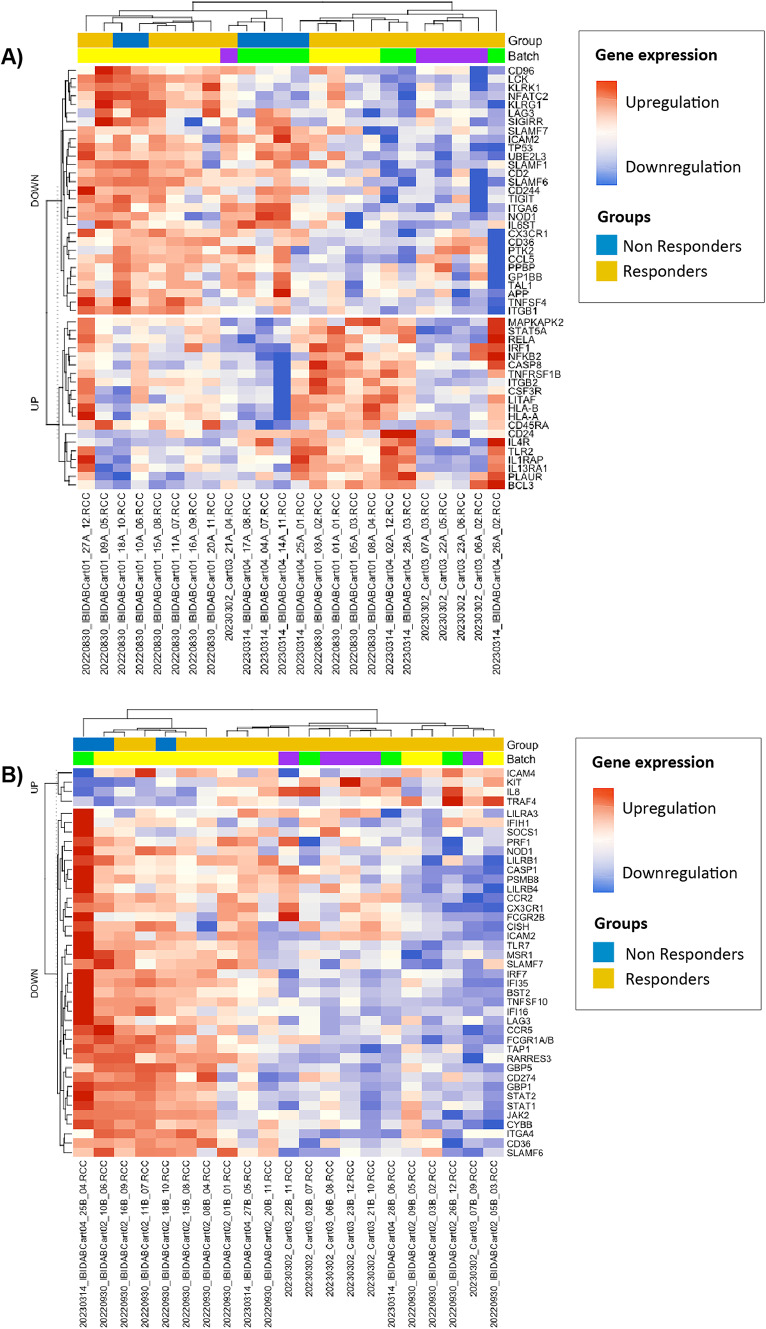
Patient stratification with the differentially expressed genes. (**A**) Differentially expressed genes at baseline (T0), (**B**) Differentially expressed genes at progression or follow up − 3 months (T1).

At T0, 49 genes displayed a significant differential expression according to treatment response, with 20 genes upregulated and 29 genes downregulated in R, as compared with NR (Supplementary Table S3). At T1, 41 genes demonstrated significant differential expression, with 4 genes upregulated and 37 genes downregulated in R (Supplementary Table S4). The heatmap in Fig. 3 illustrates the gene expression patterns at both T0 and T1, stratified by response to targeted therapy.

In the analysis comparing CR to No-CR, only one gene, *CCL11*, was significantly downregulated in the CR subgroup at T0 (*p* = 0.038).

#### Longitudinal differential gene expression

When analyzing the expression dynamics of the 49 differential expression genes at baseline, only 39 genes complied with the experimental quality standards for both timepoints, due to insufficient number of reads. No statistically significant differences were found in longitudinal differential expression from T0 to T1 in the R group nor in the NR cohort after Wilcoxon signed-rank test (Supplementary Table S5).

**Fig. 4:**
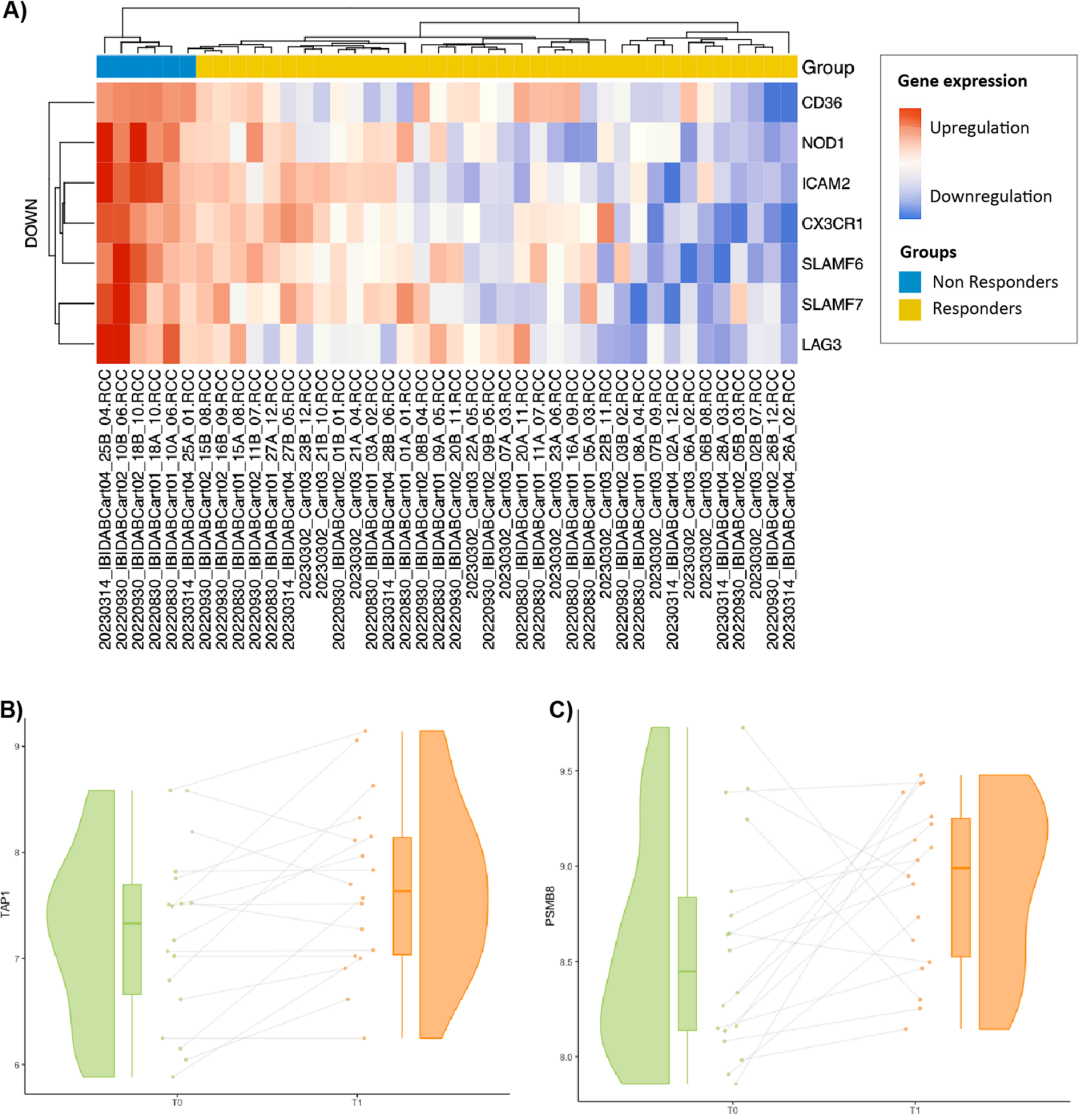
(**A**) Heatmap exhibiting 7 genes persistently downregulated in responders from baseline (T0) to progression or follow up − 3 months (T1). (**B**) Upregulation of *TAP1* (*p* = 0.012) and (**C**) *PMSB8* (*p* = 0.038) during the targeted therapy in the responder cohort.

Interestingly, 7 genes remained consistently downregulated in the R cohort at both T0 and T1: L*AG3*,* CD36*,* SLAMF7*,* NOD1*,* SLAMF6*,* CX3CR1* and *ICAM2*. In addition, two genes - *TAP1* and *PSMB8* - exhibited an increase in expression in the R group from T0 to T1, probably in response to targeted therapy. The heatmap depicting the expression of these 7 genes according to treatment response is shown in Fig. 4A, and the dynamics of *TAP1* and *PSMB8* in the R group are shown in Fig. 4B and C (Supplementary Table S6).

No significant differences were observed in the longitudinal analysis among the CR and No-CR cohorts.

## Discussion

Previous studies have linked increased alpha diversity and microbial richness in melanoma patients, particularly species from the Firmicutes phylum, with favourable ICI response and adverse effects occurrence^7,17,18^. In parallel, BRAF- or KRAS-mutated colorectal cancers have been shown to exhibit distinct microbial profiles, notably with enrichment in Fusobacteria and Firmicutes, pointing to a broader association between oncogenic mutations and gut microbiota composition^19,20^. However, the effect of BRAF/MEK-targeted therapy on GM in *BRAF*-mutated melanoma remains unexplored. These inhibitors are known to modulate immunity by enhancing CD4⁺ and CD8⁺ T-cell activity and promoting inflammatory responses within the tumor microenvironment^11,21^. Our results suggest a potential role of GM in shaping immune responses and prognosis during targeted therapy.

Our study identified a baseline enrichment of *Lachnospiraceae*, *Coriobacteriaceae*, and *Adlercreutzia* in PR to BRAF/MEK inhibitors, whereas CR exhibited increased abundance of *Prevotellaceae*, *Cerasicoccaceae*, and genus *Lawsonia*. Notably, *Lachnospiraceae*,* Coriobacteriaceae*,* Adlercreutzia*, and *Prevotellaceae* have been previously described as immune-modulating taxa, which might potentially contribute to treatment efficacy through immunoregulatory properties^6–10,22^. In contrast, to our knowledge, *Cerasicoccaceae* and *Lawsonia* have not been reported in the context of antitumor immunity, making their role in treatment response novel and warranting further investigation.

*Lachnospiraceae* is known to enhance CD8 + T cell function and produce anti-inflammatory metabolites like butyrate, as well as being associated with improved response to immunotherapy and prolonged survival^9^. Although this family has also been linked to immune-related toxicities, we did not observe such an association in our cohort, underscoring the complexity and context-dependency of microbiota–treatment interactions across therapeutic modalities^10^. Another study in ICI-treated melanoma patients undergoing microbiota transplantation found that the most enriched taxa associated with response included families *Lachnospiraceae* and *Ruminococcaceae*, as well as *Bifidobacteriaceae* and *Coriobacteriaceae*, thus further supporting the role of specific gut microbiota profiles in modulating treatment outcomes and prognosis^18^.

*Adlercreutzia*, enriched in our PR-cohort, has been described in responders to ICI in a cohort of 27 patients diagnosed with metastatic melanoma^9^. Preclinical evidence suggests that it enhances T-cell activity and that it may play a protective role in cancers like prostate cancer, where it correlates with higher S-equol levels—compounds associated with reduced cancer risk^23,24^. These findings suggest that *Adlercreutzia* may have broader immunomodulatory effects across multiple tumor types.

*Prevotellaceae*, enriched in the CR cohort, has been associated with favorable health states, including cancer-free status and regressive melanoma^25,26^. It has also been described as part of the ‘microbiome signature of health’ in the Dutch Microbiome Project, reinforcing its potential role as a beneficial modulator of host immunity^27^. However, other studies have linked this taxa to pro-inflammatory profiles and poorer clinical outcomes, highlighting the context-dependent nature of its role within the gut ecosystem^10,28,29^.

*Pasteurellaceae*, enriched in No-PR patients, includes several pathogenic species such as *Haemophilus influenzae* and *Pasteurella multocida*, which have been previously linked to poor ICI responses^8,10,12^. *Pasteurella multocida* has been shown to promote tumor growth through PI3K/Akt signaling, which might potentially contribute to resistance mechanisms to therapies targeting the MAPK pathway^30^.

*Clostridium* was enriched in both the No-CR and No-PR group. While some *Clostridium* species have been linked to better ICI responses, others such as *Clostridium spiroforme* and *Hydrogeniiclostridium mannosilyticum* have been associated with shorter PFS^8,12^, suggesting a potentially detrimental effect depending on species composition.

The relationship between GM and the incidence of adverse events remains challenging to interpret. The available evidence focuses on TRAE as a result of ICI, which may not be applicable to iBRAF/MEK given their distinct mechanisms of action^31^. *Oscillospira*, enriched in patients with grade ≥ 2 TRAE, has been negatively correlated to chronic inflammatory diseases. As a result, it has been proposed as a probiotic due to its regulatory effects on chronic inflammation^32^. In our study, the most frequent grade 2 TRAE were pyrexia, asthenia and diarrhea, suggesting increased inflammation in this context. Given the established connection of *Oscillospira* with an antiinflamatory environment, we can speculate that its presence may paradoxically contribute to these adverse effects of targeted therapy through an immune-mediated mechanism. On the other hand, taxa like *Gemmiger*, *Lachnospiraceae* and *Leuconostocaceae*, may modulate inflammation through butyrate secretion, which inhibits proinflammatory cytokine release^33,34^.

Focusing on immune-related gene expression changes, seven genes—*LAG3*,* CD36*,* SLAMF7*,* NOD1*,* SLAMF6*,* CX3CR1*, and *ICAM2*—were consistently downregulated in responders. These genes have been implicated in immune suppression, cancer progression and poor prognosis, supporting their potential as therapeutic targets^35–38^. For example, *LAG3* is an inhibitory immune checkpoint that promotes Treg activity and suppresses dendritic cell maturation, contributing to immune tolerance^35^. *CD36* has been linked to angiogenesis, apoptotic resistance, and poor outcomes; notably, its upregulation has been observed in MAPK inhibitor–resistant melanoma cells, facilitating drug tolerance^39^. *SLAMF6* and *SLAMF7* contribute to CD8 + T cell exhaustion and have been reported enriched in non-responders to ICI^37,40,41^. *NOD1* promotes the expansion of myeloid-derived suppressor cells and may influence tumor-associated disbiosis^42^. *CX3CR1* plays dual roles: it facilitates recruitment of cytotoxic immune cells (e.g., NK and CD8 + T cells), but also enhances tumor cell survival and migration via pathways such as MAPK^38,43^. *ICAM2* has similarly shown both pro- and anti-tumoral associations depending on cancer type and context^44,45^. While these genes’ downregulation in responders may suggest reduced immunosuppressive signaling, we recognize that their roles are highly context-dependent. Furthermore, since our analysis was conducted using bulk RNA from peripheral blood, it does not capture cell-type–specific expression patterns, which could influence interpretation.

Two key genes, *TAP1* and *PSMB8*, were upregulated in responders from T0 to T1. *TAP1* is involved in antigen processing and immune regulation, with its downregulation often linked to tumor immune evasion and poor prognosis, and its upregulation associated with increased ICI response^46,47^. However, in *KRAS*-mutated pancreatic cancer, Li et al. described that TAP1 overexpression increased resistance to MEK inhibitors by facilitating drug transport out of cells and promoting cancer stemness^48^. *PSMB8*, a key component of the immunoproteasome, is associated with improved prognosis in melanoma and other cancers, though conflicting evidence exists in gastrointestinal tumors^49–53^. This dual role raises questions about its influence on treatment response and potential contribution to therapy resistance in BRAF/MEK-treated melanoma.

*CCL11*, downregulated in CR at baseline, encodes a chemokine linked to Th2 inflammation and tumor progression through AKT and ERK pathway activation^54^. However, it has also been associated with improved ICI response in melanoma patients, suggesting a complex role in cancer^55^.

In our longitudinal analysis, we observed no significant shifts in GM composition or immune-related gene expression from T0 to T1. This observation is consistent with prior findings in melanoma patients treated with ICI, where microbial stability over time was also reported^12^. These results may suggest that the baseline microbiota plays a more prominent role in modulating response than dynamic changes during therapy. Nonetheless, our limited sample size may have reduced the statistical power to detect statistically significant longitudinal changes in gut microbiota or immune-related gene expression.

Our study has several limitations: the small cohort might underpower our results, the need for trained professionals and specialized equipment for GM and biomarker analysis, which may not be accessible in all hospitals, and the potential for bias due to its observational nature. The use of 16 S rRNA sequencing, unlike whole-genome shotgun metagenomics, restricts the analysis to broader taxonomic ranks such as family or genus. This limitation hampers the ability to achieve finer taxonomic resolution at the species or strain level. In addition, the low relative abundance of certain microorganisms may limit the interpretability and biological relevance of their role in treatment response and toxicity. However, emerging evidence suggests that even low-abundance microbes can exert disproportionate immunomodulatory effects within the gut ecosystem^56^. The inherent challenge lies in distinguishing whether these low-abundance taxa are genuine contributors to functional changes within the microbiota or statistical artifacts resulting from data sparsity. Our findings should be considered hypothesis-generating and will require external validation in future studies.

## Conclusion

In conclusion, our study suggests that responders to BRAF/MEK-targeted therapy in metastatic melanoma harbor a distinct gut microbiota profile enriched in previously described immune-modulating taxa such as *Lachnospiraceae*, *Coriobacteriaceae*, *Adlercreutzia* and *Prevotellaceae*. This strengthens their potential use in microbiota-modulating strategies such as the use of probiotics or in fecal microbiota transplantation. The role of the *Oscillospira* genus, enriched in patients with moderate-to-severe treatment-related adverse events, warrants further investigation to elucidate its potential contribution to adverse events occurrence.

Apart from the identification of specific GM composition, a signature of genes involved in immune reactivation was response-predictive. *LAG3*,* CD36*,* SLAMF7*,* NOD1*,* SLAMF6*,* CX3CR1*, and *ICAM2*, were downregulated in responders and represent promising actionable variants for enhancing treatment response.

Our findings contribute to the growing evidence supporting the complex relationship between microbiota composition and cancer treatment response. This opens a new field of research focusing on the influence of intestinal microbiota composition and immune-related gene expression on BRAF/MEK treatment response and adverse effects. As a result, it may pave the way for the discovery of novel biomarkers and therapeutic strategies for metastatic melanoma.

## Electronic supplementary material

Below is the link to the electronic supplementary material.

Supplementary Material 1

## Data Availability

The datasets generated and analyzed during the current study are available in the NCBI repository, accession number PRJNA1272598, and in the Gene Expression Omnibus (GEO) repository, accession number GSE299194.

## Abbreviations

AJCC: American Joint Committee on Cancer
CR: Complete response
CTCAE: Common Terminology Criteria for Adverse Events
GM: Gut microbiota
HR: Hazard ratio
ICI: Immune checkpoint inhibitors
iBRAF/MEK: BRAF/MEK inhibitors
LEfSe: Linear discriminant analysis effect size
NMDS: Non-metric multidimensional scaling
NR: Non-Responders
No-CR: No complete response
No-PR: No partial response
PD: Progressive disease
PFS: Progression-free survival
PR: Partial response
R: Responders
RECIST: Response evaluation criteria in solid tumors
rRNA: ribosomal RNA
RUVg: Remove unwanted variation using control genes
SD: Stable disease
TKI: Tyrosine kinase inhibitors
TME: Tumor microenvironment
TRAE: Treatment-related adverse events
OS: Overall survival
OTUs: Operational Taxonomic Units
95%CI: 95% Confidence interval
