# Supplementary material for: Association of gut microbiota and immune gene expression with response to targeted therapy in BRAF mutated melanoma
Source: Sci Rep. 2025 Jul 14;15:25430. doi: 10.1038/s41598-025-11054-2 (PMC12259845; doi:10.1038/s41598-025-11054-2)
Supplement: Supplementary file 1 — Supplementary Material 1 [file 41598_2025_11054_MOESM1_ESM.pdf]

Gut Microbiota and Immune Gene Expression: Association With Treatment Response in Melanoma Patients on Targeted Therapy

Mora Guardamagna [1,2,3], Miguel-Angel Berciano-Guerrero [2, 4*], Rocío Lavado-Valenzuela [1 5], Édouard Auclin [6], Juan Luis Onieva Zafra[1], Isaac Plaza Andrades [1], Javier Oliver [1], Alicia Garrido-Aranda [1 5], Elisabeth Perez-Ruiz [4], Martina Álvarez [5 7], María Carmen Ocaña [5], María Isabel Queipo-Ortuño [1,8,] Isabel Barragán+ [1,10], Antonio Rueda-Dominguez+ [1,2]

[1] Virgen de la Victoria University Hospital, Instituto de Investigación Biomédica de Málaga y Plataforma en Nanomedicina-IBIMA Plataforma BIONAND, 29010 Málaga, Spain

[2] Department of Medicine and Dermatology, Medical School University of Málaga. Campus Teatinos, Blvr. Louis Pasteur, 32, 29010 Málaga, Spain

[3] Department of Cancer Medicine - Institut Gustave Roussy, Villejuif, France.

[4] Regional University Hospital, Instituto de Investigación Biomédica de Málaga y Plataforma en Nanomedicina-IBIMA Plataforma BIONAND, 29010 Málaga, Spain

[5] Cancer Molecular Biology Laboratory, CIMES, Malaga, Spain.

[6] Institut Bergonié, Bordeaux, France

[7] Pathological Anatomy Department. Medical School University of Málaga. Campus Teatinos, Blvr. Louis Pasteur, 32, 29010 Málaga, Spain

[8] Department of Surgical Specialties, Biochemical and Immunology. Faculty of Medicine, University of Málaga. 29071 Malaga, Spain

[9] Group of Pharmacoepigenetics, Department of Physiology and Pharmacology, Karolinska Institute, 171 65, Stockholm, Sweden.

[10] Group of Translational Research in Cancer Immunotherapy (B-05), Medical Oncology Unit of Virgen de la Victoria Hospital, Instituto de Investigación Biomédica de Málaga y Plataforma en Nanomedicina–IBIMA Plataforma Bionand, 29010, Malaga, Spain.

***** Correspondence: mangel.berciano.sspa@juntadeandalucia.es

+ Equal contribution

Table S1. Univariate analysis in the gut microbiota protocol cohort (n=20). 95%CI=95% Confidence interval, ECOG-PS=Eastern Cooperative Oncology Group Performance Status, TT=Targeted treatment, TRAE=Treatment-related adverse event, ANC=Absolute neutrophil count, LDH=Lactate dehydrogenase, ULN=Upper limit of normal, NLR=Neutrophil-to-lymphocyte ratio, dNLR=Derived NLR.

Univariate analysis (N=20)	Overall Survival			Progression Free Survival		
**Clinical Characteristics**	**HR**	**95%CI**	**P**	**HR**	**95%CI**	**P**
**Age**	1.00	0.95-1.07	0.77	0.98	0.92-1.04	0.42
**Sex, male**	0.72	0.16-3.29	0.67	0.57	0.16-1.97	0.37
**ECOG PS 0 vs 1-2**	5.17	0.61-43.60	0.13	0.64	0.19-2.13	0.64
**Prior adjuvant treatment**	0.36	0.07-1.92	0.23	0.28	0.07-1.11	0.07
**Antibiotics 30 days previous TT**	3.99	0.66-24.1	0.13	1.60	0.32-7.99	0.57
**Antibiotics during TT**	1.14	0.21-6.22	0.88	1.03	0.29-3.58	0.97
**Corticosteroids 30 days previous TT**	1.26	0.24-6.55	0.78	0.64	0.14-2.97	0.57
**Corticosteroids during TT**	3.75	0.45-31.26	0.22	1.83	0.48-6.93	0.37
**Laboratory Variables**						
**ANC ≥7500/mL**	1.28	0.24-6.84	0.77	0.32	0.04-2.55	0.28
**LDH≥ULN**	1.13	0.24-5.27	0.87	1.44	0.42-4.93	0.57
**dNLR≥3**	1.43	0.30-6.83	0.65	1.13	0.29-4.31	0.83
**NLR≥5**	0.84	0.15-4.79	0.85	1.32	0.34-5.03	0.68
**Adverse Events to TT**						
**TRAE ≥2**	0.84	0.18-3.87	0.82	1.79	0.53-5.97	0.34
**Diarrhea**	0.50	0.09-2.66	0.42	0.57	0.16-2.04	0.39
**Pyrexia**	1.64	0.36-7.52	0.53	1.10	0.33-3.65	0.87
**Asthenia**	0.84	0.18-3.98	0.86	0.66	0.19-2.26	0.51
**Nausea/Vomiting**	0.95	0.18-4.97	0.95	0.82	0.21-3.16	0.78

Table S2. Multivariate analysis in the gut microbiome protocol cohort (n=20). OS=Overall Survival, PFS= Progression-Free Survival, TT= Targeted treatment, TRAE= Treatment-related adverse event, HR=Hazard ratio, 95%CI=95% Confidence interval.

	**OS**			**PFS**		
**Multivariate analysis (N=20)**	**HR**	**95%CI**	**P**	**HR**	**95%CI**	**P**
**Antibiotics during TT**	2.79	0.29-26.49	0.37	1.41	0.30-6.55	0.66
**TRAE grade ≥ 2**	1.57	0.28-8.59	0.60	1.61	0.44-5.91	0.47
Lachnospiraceae	0.99	0.99-1.00	0.058	1.00	0.99-1.00	0.33
Coriobacteriaceae	0.99	0.99-1.00	0.17	1.00	0.99-1.00	0.67
Adlercreutzia	1.01	0.96-1.06	0.55	1.01	0.99-1.02	0.41

Family level - family repartition

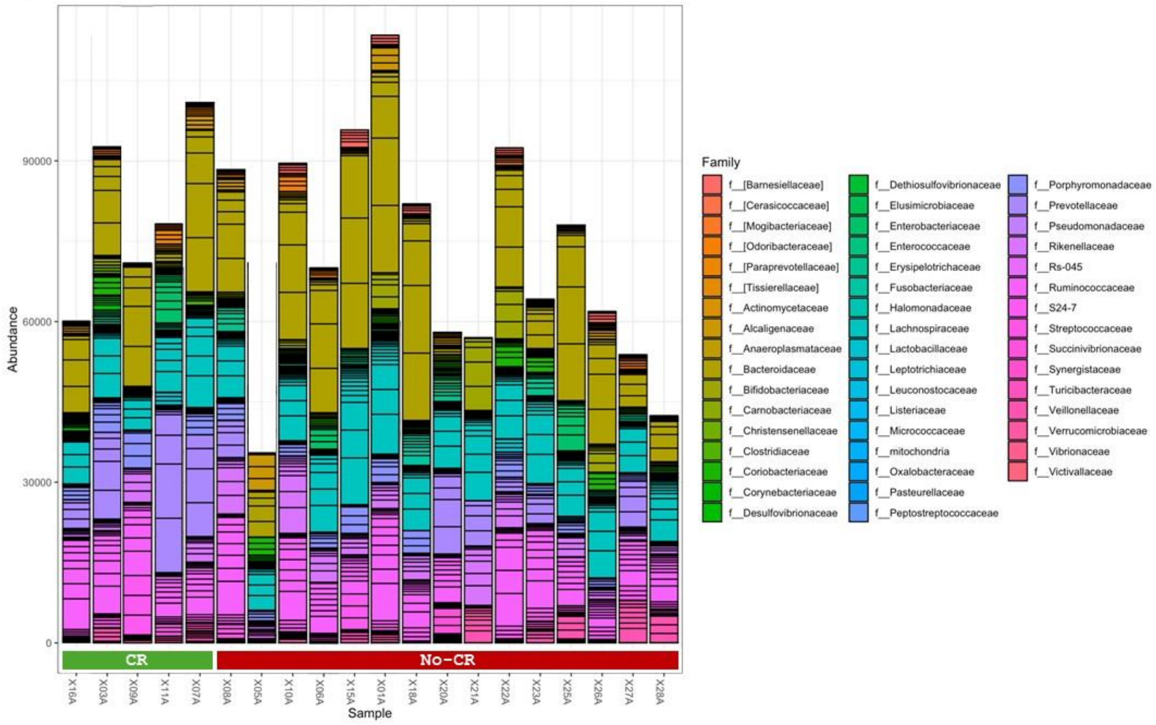

Genus level - genus repartition

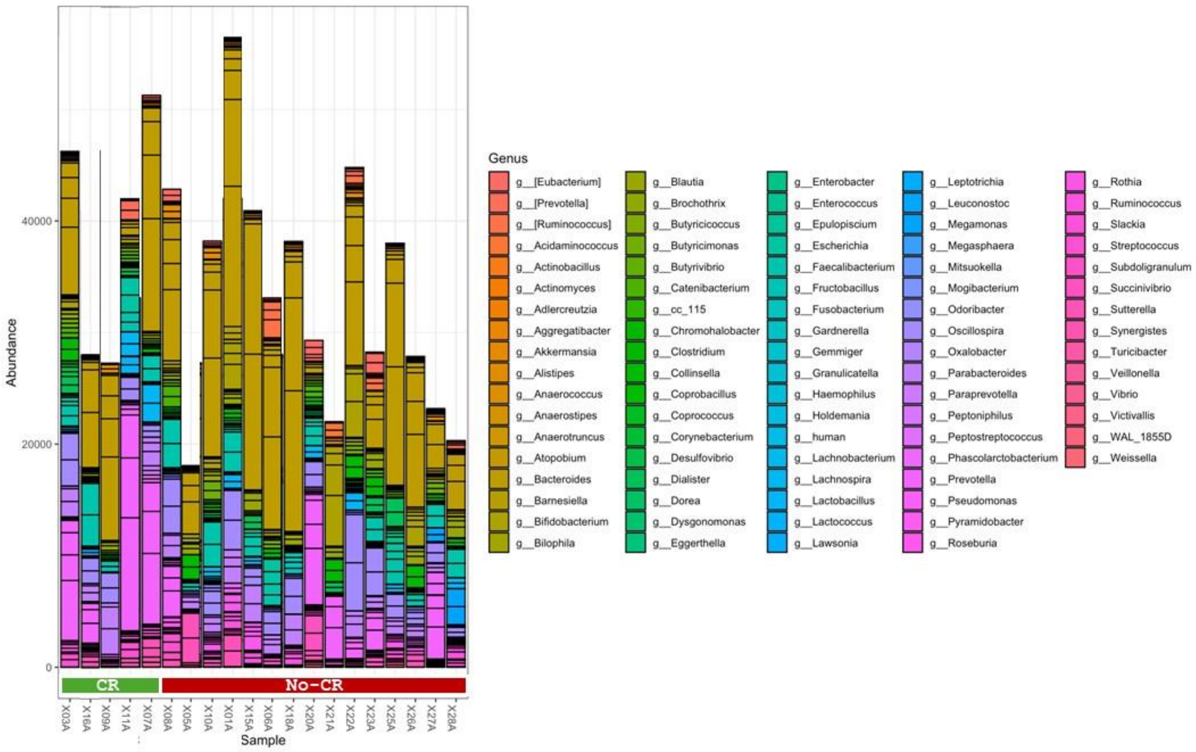

Figure S1. Relative abundances according to family and genus taxonomy level. CR=Complete response, No-CR=No complete response

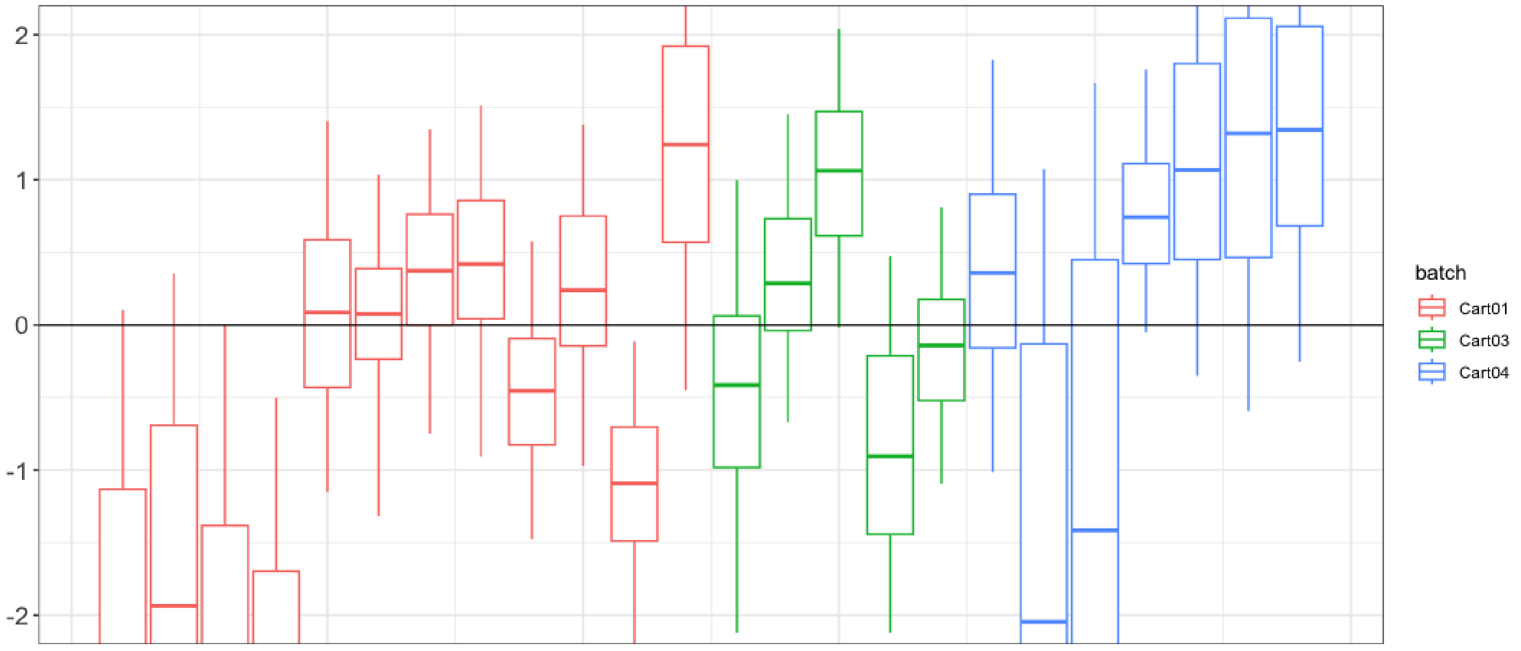

Figure S2. Gene expression variability in twenty-four patients.

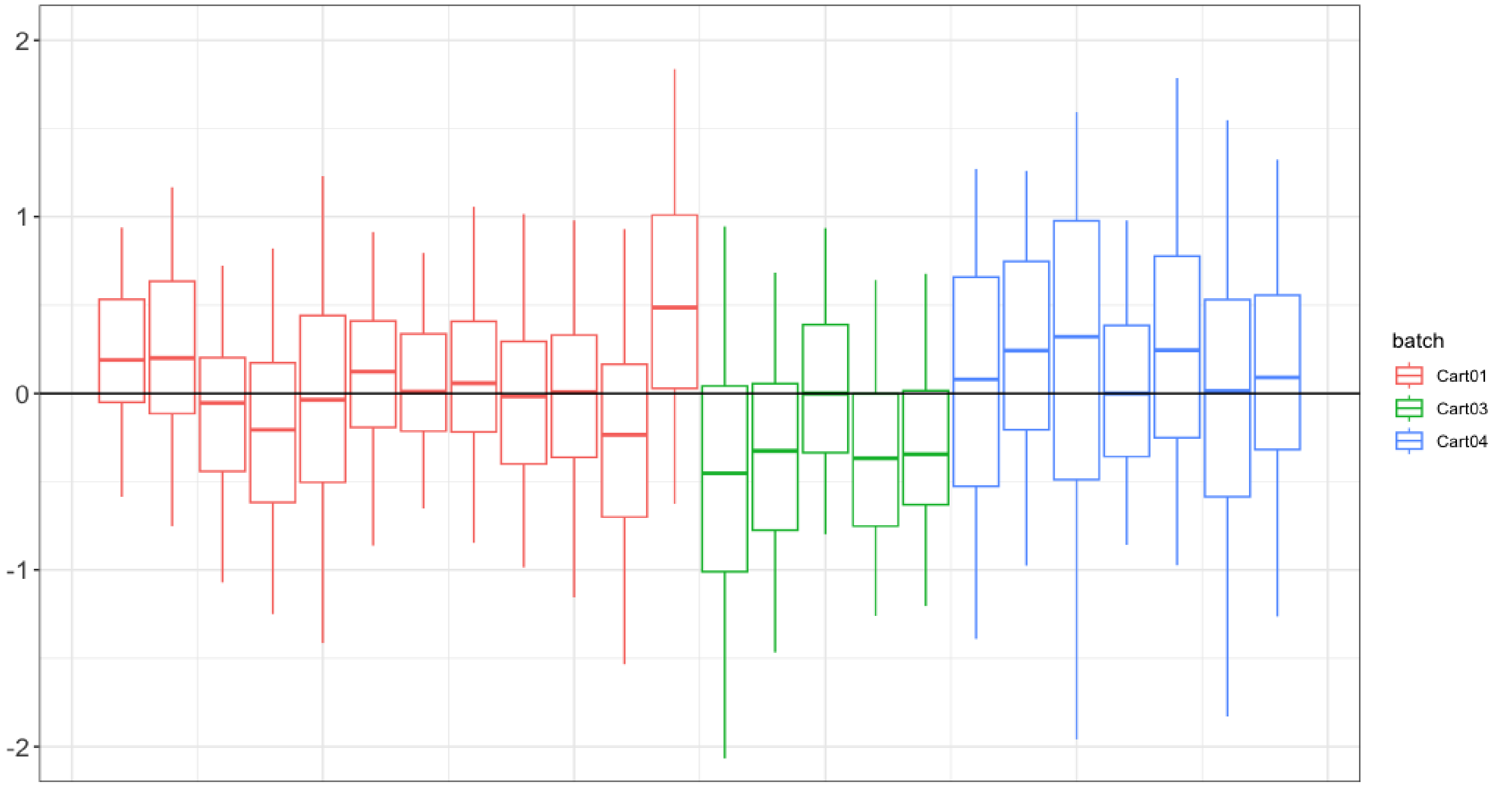

Figure S3. Gene expression variability in twenty-four patients after normalization with RUVg.

Genes at Basal Time	logFC	pvalue
LAG3	-0,78	0,041
ITGA6	-0,93	0,011
TP53	-0,75	0,017
KLRK1	-0,77	0,049
RELA	0,77	0,011
MAPKAPK2	0,89	0,0062
IL4R	0,63	0,035
TAL1	-1,1	0,0091
CD36	-1,2	0,017
CD45RA	0,69	0,044
LITAF	0,81	0,048
NFATC2	-0,6	0,044
TIGIT	-0,87	0,023
NFKB2	1,1	0,0097
HLA-A	0,77	0,0078
STAT5A	0,56	0,018
LCK	-0,74	0,049
BCL3	1,2	0,0022
SLAMF7	-0,94	0,025
PTK2	-0,77	0,0069
NOD1	-0,64	0,026
UBE2L3	-0,65	0,035
CD24	1	0,024
TNFRSF1B	0,88	0,013
CASP8	0,68	0,021
TNFSF4	-0,81	0,036
ITGB2	0,73	0,049
GP1BB	-1,1	0,018

Genes at Basal Time	logFC	pvalue
IL1RAP	0,63	0,042
HLA-B	0,7	0,01
CD244	-0,57	0,045
CD96	-0,61	0,039
TLR2	0,66	0,049
CD2	-0,84	0,009
SLAMF6	-1	0,0043
CCL5	-1,3	0,00092
ITGB1	-0,7	0,014
SIGIRR	-0,57	0,042
CX3CR1	-1,5	0,0022
APP	-0,6	0,048
PPBP	-1,5	0,0088
PLAUR	0,87	0,015
IL13RA1	0,77	0,044
CSF3R	1,2	0,018
SLAMF1	-0,81	0,031
ICAM2	-0,66	0,022
IL6ST	-0,55	0,039
IRF1	0,72	0,036
KLRG1	-1,1	0,039

Table S3. Genes at T0 with corresponding log-fold change and p-value.

Genes at T1	logFC	pvalue
KIT	0,85	0,041
LAG3	-1,5	0,0038
CD36	-0,93	0,039
CCR5	-1,5	0,0015
CISH	-1	0,041
PSMB8	-0,69	0,047
IRF7	-1,4	0,011
GBP5	-1,7	0,02
SLAMF7	-0,95	0,038
ITGA4	-0,64	0,049
IFI16	-1,3	0,0034
NOD1	-0,8	0,016
GBP1	-2,3	0,0036
MSR1	-1,5	0,0089
JAK2	-1,1	0,018
IFI35	-1,7	0,0072
FCGR1A/B	-1,6	0,015
CD274	-1,7	0,045
SOCS1	-1	0,047
SLAMF6	-0,88	0,021
STAT1	-1,2	0,022
STAT2	-1,4	0,0026
CX3CR1	-1,9	0,036
TRAF4	1,2	0,017
ICAM4	0,81	0,038
TAP1	-1,1	0,009
CYBB	-0,93	0,047
FCGR2B	-1,5	0,047

Genes at T1	logFC	pvalue
TNFSF10	-2,1	0,0022
LILRA3	-1,6	0,035
PRF1	-1	0,038
TLR7	-1,4	0,0046
RARRES3	-0,95	0,013
CCR2	-1,5	0,045
IFIH1	-1,1	0,038
LILRB1	-1,1	0,038
IL8	2,8	0,0065
BST2	-1,4	0,0047
CASP1	-1,4	0,02
ICAM2	-0,77	0,041
LILRB4	-1,2	0,021

Table S4. Genes at T1 with corresponding log-fold change and corresponding p-value.

Changes in Genes from T0 to T1	P-value Responders	P-value Non Responders
ITGA6	0,129737854003906	1
TP53	0,83172607421875	0,5
KLRK1	0,181465148925781	0,5
RELA	0,865043640136719	0,75
MAPKAPK2	0,346549987792969	0,25
TAL1	1	0,5
CD36	0,733726501464844	1
CD45RA	0,0897674560546875	0,75
NFATC2	0,4171142578125	0,75
TIGIT	0,266712256717957	0,25
NFKB2	0,442298889160156	0,75
STAT5A	0,442298889160156	0,5
LCK	0,246208190917969	0,5
BCL3	0,898574829101563	0,75
SLAMF7	0,2645263671875	0,25
PTK2	0,609458923339844	0,75
NOD1	0,2645263671875	0,5
UBE2L3	0,369216918945313	1
TNFRSF1B	0,167350769042969	0,75
CASP8	0,303794860839844	0,75
IL6ST	0,550872802734375	0,75
IRF1	0,246208190917969	0,25
KLRG1	0,7987060546875	0,25
ITGB2	0,550872802734375	0,75
GP1BB	0,966117858886719	0,25
HLA-B	0,83172607421875	0,75
CD244	0,966117858886719	0,75
CD96	0,766029357910156	1
CD2	0,966117858886719	0,25

Changes in Genes from T0 to T1	P-value Responders	P-value Non Responders
SLAMF6	0,7987060546875	0,25
CCL5	0,154045104980469	1
ITGB1	0,212142944335938	0,75
SIGIRR	0,176945075573857	0,75
CX3CR1	0,639694213867188	0,25
APP	0,154045104980469	0,25
PPBP	0,701881408691406	0,5
CSF3R	0,0814285278320313	1
SLAMF1	0,163385074154965	0,5
ICAM2	0,538293635901243	0,25

Table S5. Differential expression of immune-associated genes from T0 to T1.

Table S6. Differential expression in immune-associated genes at T1 from T0.

Changes in genes at T1 from T0	p-value Responders	p-value Non Responders
CD36	0,733726501464844	1
CCR5	0,442298889160156	0,25
CISH	0,246208190917969	0,25
PSMB8	0,0384902954101563	0,5
SLAMF7	0,2645263671875	0,25
ITGA4	0,932281494140625	0,25
NOD1	0,2645263671875	0,5
JAK2	0,966117858886719	0,25
SLAMF6	0,7987060546875	0,25
STAT1	0,303794860839844	0,25
CX3CR1	0,639694213867188	0,25
TRAF4	0,102399198063522	0,5
ICAM4	0,346549987792969	1
TAP1	0,0121459523645502	0,25
PRF1	0,141517639160156	0,25
CYBB	0,639694213867188	0,25
RARRES3	0,212142944335938	0,25
CCR2	0,442298889160156	0,25
LILRB1	0,924572011837841	0,5
ICAM2	0,538293635901243	0,25
